# Global Exponential Stability of Almost Periodic Solution for Neutral-Type Cohen-Grossberg Shunting Inhibitory Cellular Neural Networks with Distributed Delays and Impulses

**DOI:** 10.1155/2016/6508734

**Published:** 2016-03-30

**Authors:** Lijun Xu, Qi Jiang, Guodong Gu

**Affiliations:** ^1^School of Mathematics and Computer Science, Panzhihua University, Panzhihua, Sichuan 617000, China; ^2^The Academy of Agriculture and Forestry Sciences of Panzhihua City, Panzhihua, Sichuan 617000, China

## Abstract

A kind of neutral-type Cohen-Grossberg shunting inhibitory cellular neural networks with distributed delays and impulses is considered. Firstly, by using the theory of impulsive differential equations and the contracting mapping principle, the existence and uniqueness of the almost periodic solution for the above system are obtained. Secondly, by constructing a suitable Lyapunov functional, the global exponential stability of the unique almost periodic solution is also investigated. The work in this paper improves and extends some results in recent years. As an application, an example and numerical simulations are presented to demonstrate the feasibility and effectiveness of the main results.

## 1. Introduction

It is well known that shunting inhibitory cellular neural networks (SICNNs) [1] have many applications in psychophysics, speech, perception, robotics, adaptive pattern recognition, vision, and image processing. Therefore, the stability problem for SICNNs has been one of the most active areas of research and there exist some results on the existence and stability of periodic and almost periodic solutions for the SICNNs with delays [2–11]. In applications, almost periodic oscillation is more accordant with fact [12–14]. Therefore, there are some good results on the existence and global exponential stability of almost periodic solutions for SICNNs [3–9].

The Cohen-Grossberg neural network (CGNN) [15] is a kind of important neural network described as follows:

(1) x˙i=−aixibixi−∑j=1ncijfjxj−Ii,i=1,2,…,n,

where *n* ≥ 2 is the number of neurons in the network, *x*_*i*_ = *x*_*i*_(*t*) denotes the state variable associated with the *i*th neuron, *a*_*i*_ represents an amplification function, and *b*_*i*_ is an appropriately behaved function such that the solution of the above model remains bounded. The *n* × *n* connection matrix *A* = (*c*_*ij*_) tells how the neurons connected in the network, and the activation function *f*_*j*_ shows how the *j*th neuron reacts to the input; *I*_*i*_ represents external input at the time *t*. Cohen-Grossberg neural networks have been extensively studied because of their immense potentials of application perspective in different areas such as pattern recognition, optimization, signal, and image processing. In addition, experiments show that time delays can affect the stability of neural networks and lead to some other dynamical behaviors, such as periodic or almost periodic oscillation, bifurcation, and chaos. Hence, they have been the object of intensive analysis by numerous authors and some good results on the existence and global exponential stability of periodic and almost periodic solutions for Cohen-Grossberg neural networks with delays have been obtained [16–28].

On the other hand, the states of many processes and phenomena studied in optimal control, biology, mechanics, biotechnologies, medicine, electronics, economics, and so forth are often subject to instantaneous perturbations and experience abrupt changes at certain moments of time. The duration of these changes is very short and negligible in comparison with the duration of the process considered and can be thought of as “momentary” changes or as impulses. Systems with short-term perturbations are often naturally described by impulsive differential equations. Owing to its theoretical and practical significance, the theory of impulsive differential equations has undergone a rapid development in the last couple of decades [29–33].

Stimulated by the above reasons, Yang [26] considered the following Cohen-Grossberg SICNNs with distributed delays, which has a more general and complicated dynamics than SICNNs. By using Schaeffer's theorem and constructing suitable Lyapunov functional, he obtained the existence and global exponential stability of periodic solution of the following impulsive Cohen-Grossberg SICNNs with delays: 
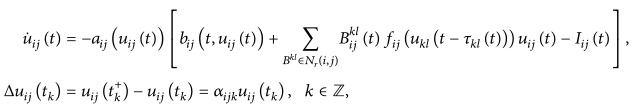
(2)where Δ*u*_*ij*_(*t*_*k*_) = *u*_*ij*_(*t*_*k*_^+^) − *u*_*ij*_(*t*_*k*_) is the impulse at moment *t*_*k*_ and *t*_*k*_ < *t*_*k*+1_, lim_*k*→+*∞*_ *t*_*k*_ = +*∞*, *B*_*ij*_ denotes the cell at the (*i*, *j*) position of the lattice, the *r*-neighborhood *N*_*r*_(*i*, *j*) of *C*_*ij*_ is

(3) Nri,j=Bijkl:max⁡k−i,l−j≤r,  1≤k≤m,  1≤l≤n,

*u*_*ij*_ is the activity of the cell *B*_*ij*_, *I*_*ij*_ is the external input to *B*_*ij*_, *a*_*ij*_ and *b*_*ij*_ represent an amplification function and an appropriately behaved function, respectively, nonnegative function *B*_*ij*_^*kl*^ is the connection or coupling strength of postsynaptic activity of the cell transmitted to the cell *B*_*ij*_, and the activity function *f*_*ij*_(*u*_*kl*_) is continuous function representing the output or firing rate of the cell *B* ^*kl*^, where *i* = 1,2,…, *m* and *j* = 1,2,…, *n*.

In addition, owing to the complicated dynamic properties of the neural cells in the real world, the existing neural network models in many cases cannot characterize the properties of a neural reaction process precisely. It is natural that systems will contain some information about the derivative of the past state to further describe and model the dynamics for such complex neural reactions. This new type of neural networks is called neutral neural networks or neural networks of neutral type. The motivation for us to study neural networks of neutral type comes from three aspects. First, based on biochemistry experiments, neural information may transfer across chemical reactivity, which results in a neutral-type process. Second, in view of electronics, it has been shown that neutral phenomena exist in large-scale integrated (LSI) circuits. Last, the key point is that cerebra can be considered as a super LSI circuit with chemical reactivity, which reasonably implies that the neutral dynamic behaviors should be included in neural dynamic systems [34–37].

In the literatures, although there are numerous results on the existence and stability of neural networks with delays, the problem of global exponential stability of almost periodic solution for neutral-type Cohen-Grossberg SICNNs has not been fully investigated. And, in most situations, delays are in fact unbounded and a neural network usually has a spatial nature due to the presence of various parallel pathways. That is, the entire history affects the present, so distributed delays are more suitable to practical neural networks (see [9, 10, 17, 18, 23–25]).

Therefore, in this paper, we consider the following neutral-type Cohen-Grossberg SICNNs with distributed delays:
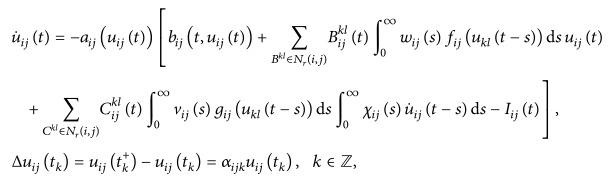
(4)where the kernel functions *w*_*ij*_, *v*_*ij*_, *χ*_*ij*_ : [0, +*∞*)→[0, +*∞*) are continuous with ∫_0_^*∞*^ *w*_*ij*_(*s*)d*s* = ∫_0_^*∞*^ *v*_*ij*_(*s*)d*s* = ∫_0_^*∞*^ *χ*_*ij*_(*s*)d*s* = 1 and satisfy

(5) ∫0∞eξswijsds=pijξ,∫0∞eξsvijsds=qijξ,∫0∞eξsχijsds=oijξ,

where *p*_*ij*_, *q*_*ij*_, and *o*_*ij*_ are continuous functions in [0, *ζ*]  (*ζ* > 0) and *p*_*ij*_(0) = *q*_*ij*_(0) = *o*_*ij*_(0) = 1, where *i* = 1,2,…, *m* and *j* = 1,2,…, *n*.

The state of electronic networks is often subject to instantaneous perturbations and experiences abrupt changes at certain instants, which may be caused by switching phenomenon, frequency change, or other sudden noise that exhibit impulsive effects [30, 38]. For example, according to Arbib [39] and Haykin [40], when a stimulus from the body or the external environment is received by receptors the electrical impulses will be conveyed to the neural net and impulsive effects arise naturally in the net. An artificial electronic system, such as neural network, is often subject to impulsive perturbation; the abrupt changes in the voltages produced by faulty circuit elements are exemplary of impulse phenomena, which can affect the dynamical behaviors of the system. Besides, in contrast to the retarded differential systems, the neutral differential systems in which time delays appear explicitly in the state velocity vector can be applied to describe more complicated nonlinear engineering and bioscience models, for example, population ecology [41], the distributed networks with lossless transmission lines [42, 43], chemical reactors [44], and partial element equivalent circuits in very large-scale integration (VLSI) system [45]. Therefore, neutral delays and impulses can heavily affect the dynamical behaviors of the networks, and thus it is necessary to investigate both effects of neutral delays and impulses on the dynamics of neural networks.

The main purpose of this paper is to establish some new sufficient conditions on the existence, uniqueness, and global exponential stability of almost periodic solution of neutral-type Cohen-Grossberg SICNNs (4). First, by using the almost periodic theory of impulsive differential equations [31] and the contracting mapping principle, the existence and uniqueness of almost periodic solution of system (4) are considered. Further, by constructing a suitable Lyapunov functional, the global exponential stability of system (4) is also investigated. The main results in this paper compensate for the deficiency in papers [21–25] and extend the main results in [3–8, 26] (see Remarks 9, 10, 12, and 13).

The organization of this paper is as follows. In Section 2, we give some basic definitions and necessary lemmas which will be used in later sections. In Sections 3 and 4, by using the contracting mapping principle and constructing suitable Lyapunov functional, we obtain some sufficient conditions ensuring existence, uniqueness, and global exponential stability of almost periodic solution of system (4). Finally, an example and numerical simulations are given to illustrate that our results are feasible.

## 2. Preliminaries

Now, let us state the following definitions and lemmas, which will be useful in proving our main result.

Since the solution of system (4) is a piecewise continuous function with points of discontinuity of the first kind *t*_*k*_, *k* ∈ *ℤ*, we adopt the following definitions for almost periodicity.

**Definition 1 (see [31]).**

The set of sequences {*t*_*k*_^*j*^}, *t*_*k*_^*j*^ = *t*_*k*+*j*_ − *t*_*k*_, where *k* ∈ *ℤ*, *j* ∈ *ℤ*, and {*t*_*k*_} ∈ *𝕀*, is said to be uniformly almost periodic if for arbitrary *ϵ* > 0 there exists a relatively dense set of *ϵ*-almost periods common for any sequences.

By *𝕀*, *𝕀* = {{*t*_*k*_} ∈ *ℝ* : *t*_*k*_ < *t*_*k*+1_, *k* ∈ *ℤ*, lim_*k*→±*∞*_ *t*_*k*_ = ±*∞*}, we denote the set of all sequences that are unbounded and strictly increasing. Let *Ω* ⊂ *ℝ* and *Ω* ≠ *∅*; the following notations are introduced.

*PC*(*ξ*_0_) is the space of all functions *ϕ* : (−*∞*, *ξ*_0_] → *Ω* having points of discontinuity at *μ*_1_, *μ*_2_,…∈(−*∞*, *ξ*_0_] of the first kind and left continuous at these points.

For *J* ⊂ *ℝ*, *PC*(*J*, *ℝ*) is the space of all piecewise continuous functions from *J* to *ℝ* with points of discontinuity of the first kind *t*_*k*_, at which it is left continuous; *PC* ^1^(*J*, *ℝ*) is the space of all continuously differentiable functions from *J* to *ℝ* except the points *t*_*k*_.

Let *ϕ*_*ij*_ ∈ *PC*(0); denote by *u*_*ij*_(*t*) = *u*_*ij*_(*t*; 0, *ϕ*_*ij*_), *u*_*ij*_ ∈ *Ω*, the solution of system (4) satisfying the initial conditions

(6) uijs=ϕijs,u˙ijs=ϕ˙ijs,∀s∈−∞,0,  ϕij∈PC1−∞,0,R,

where *i* = 1,2,…, *m* and *j* = 1,2,…, *n*.

**Definition 2 (see [31]).**

The function *φ* ∈ *PC*(*ℝ*, *ℝ* ^*m*×*n*^) is said to be almost periodic, if the following hold:

(1) The set of sequences {*t*_*k*_^*j*^}, *t*_*k*_^*j*^ = *t*_*k*+*j*_ − *t*_*k*_, where *k* ∈ *ℤ*, *j* ∈ *ℤ*, and {*t*_*k*_} ∈ *𝕀*, is uniformly almost periodic.
(2) For any *ϵ* > 0 there exists a real number *δ* > 0 such that if the points *t*′ and *t*′′ belong to one and the same interval of continuity of *φ*(*t*) and satisfy the inequality |*t*′ − *t*′′| < *δ*, then ‖*φ*(*t*′) − *φ*(*t*′′)‖ < *ϵ*.
(3) For any *ϵ* > 0 there exists a relatively dense set *T* such that if *η* ∈ *T*, then ‖*φ*(*t* + *η*) − *φ*(*t*)‖ < *ϵ* for all *t* ∈ *ℝ* satisfying the condition |*t* − *t*_*k*_ | >*ϵ*, *k* ∈ *ℤ*. The elements of *T* are called *ϵ*-almost periods.

**Lemma 3 (see [31]).**

Let {*t*_*k*_} ∈ *𝕀*. Then there exists a positive integer *N* such that, on each interval of length 1, one has no more than *N* elements of the sequence {*t*_*k*_}; that is,

(7) $$\begin{array}{c} i\left(\;s,t\;\right)\leq N\left(\;t-s\;\right)+N, \end{array}$$

where *i*(*s*, *t*) is the number of the points *t*_*k*_ in the interval (*s*, *t*).

**Definition 4 .**

The almost periodic solution *x* = {*x*_*ij*_} of system (4) with the initial value *φ* = {*φ*_*ij*_} is said to be globally exponentially stable, if there exist constants *ω* > 0 and *M* ≥ 1, for any solution *y* = {*y*_*ij*_} of system (4) with initial value *ϕ* = {*ϕ*_*ij*_} such that

(8) $$\begin{array}{c} \left\|\;x-y\;\right\|\leq M{\left\|\;\varphi -\phi \;\right\|}_{\infty }e^{-\omega t},\;\forall t>0, \end{array}$$

where

(9) φ−ϕ∞≔∑i=1m ∑j=1n sups∈−∞,0⁡φijs−ϕijs+φ˙ijs−ϕ˙ijs.

Throughout this paper, we set

(10) $$\begin{array}{c} x=\left\{\;x_{ij}\;\right\}={\left(\;x_{11},\ldots ,x_{1n},\ldots ,x_{m1},\ldots ,x_{mn}\;\right)}^{T}\in R^{m\times n}. \end{array}$$

For *x* = {*x*_*ij*_} ∈ *ℝ* ^*m*×*n*^, we define the norm ‖*x*‖ = max_(*i*,*j*)_sup_*s*∈*ℝ*_⁡|*x*_*ij*_(*s*)|.

We list some assumptions which will be used in this paper.

$(H_{1}$) *B*_*ij*_^*kl*^, *C*_*ij*_^*kl*^, and *I*_*ij*_ are continuous almost periodic functions, where *i*, *k* = 1,2,…, *m* and *j*, *l* = 1,2,…, *n*.
($H_{2}$) *a*_*ij*_ ∈ *C*(*ℝ*) and there exists positive constant ${\bar{a}}_{ij}$ such that $0<a_{ij}(t)\leq {\bar{a}}_{ij}$, ∀*t* ∈ *ℝ*, where *i* = 1,2,…, *m* and *j* = 1,2,…, *n*.
($H_{3}$) *b*_*ij*_ ∈ *C*(*ℝ* ^2^, *ℝ*) is almost periodic about the first argument, *b*_*ij*_(*t*, 0) = 0, and there are positive constants ${\underline{\theta }}_{ij}$, ${\bar{\theta }}_{ij}$, ${\underline{\sigma }}_{ij}$, and ${\bar{\sigma }}_{ij}$ such that
  (11) θ_ij≤aijubijt,u−aijvbijt,vu−v≤θ¯ij,aijubijt,uu−aijvbijt,vv≤σ¯iju−v,
 ∀(*t*, *u*), (*t*, *v*)∈(*ℝ*, *ℝ*), where *i* = 1,2,…, *m* and *j* = 1,2,…, *n*.
($H_{4}$) *f*_*ij*_, *g*_*ij*_ ∈ *C*(*ℝ*), *f*_*ij*_(0) = *g*_*ij*_(0) = 0, and there exist positive constants *L*_*ij*_^*f*^, *L*_*ij*_^*g*^, and *L*_*ij*_^*a*^ such that
  (12) fiju−fijv≤Lijfu−v,giju−gijv≤Lijgu−v,aiju−aijv≤Lijau−v,
 ∀*u*, *v* ∈ *ℝ*, where *i* = 1,2,…, *m* and *j* = 1,2,…, *n*.
($H_{5}$) There exist positive constants *M*_*ij*_^*f*^ and *M*_*ij*_^*g*^ such that
  (13) fiju≤Mijf,giju≤Mijg,
 ∀*u* ∈ *ℝ*, where *i* = 1,2,…, *m* and *j* = 1,2,…, *n*.
($H_{6}$) The set of sequences {*t*_*k*_^*j*^}, *t*_*k*_^*j*^ = *t*_*k*+*j*_ − *t*_*k*_, where *k* ∈ *ℤ*, *j* ∈ *ℤ*, and {*t*_*k*_} ∈ *𝕀*, is uniformly almost periodic and there exists *θ* > 0 such that inf_*k*∈*ℤ*_⁡*t*_*k*_^1^ = *θ* > 0.
($H_{7}$) The sequence {*α*_*ijk*_} is almost periodic.

**Remark 5 .**

By (*H*_4_) and (*H*_5_) and Theorem  1.4 in [31] and Theorem  6.1.1 in [46], we can easily obtain that system (4) has a unique solution on *ℝ*.

Let

(14) X=u∈PCR,Rm×n:u  is  differential  on  R∖tk,u  and  u˙  are  almost  periodic

with the norm ${\left\|u\right\|}_{𝕏}=\max\left\{\left\|u(s)\right\|,\left\|\dot{u}(s)\right\|\right\}$. Then *𝕏* is a Banach space with the norm ‖·‖_*𝕏*_.

∀*φ* ∈ *𝕏*, consider the following auxiliary systems:

(15) u˙ijt=−aijφijtbijt,φijtφijtuijt−aijφijt·∑Ckl∈Nqi,jBijklt∫0+∞wijsfijφklt−sds+∑Ckl∈Nri,jCijklt∫0+∞vijsgijφklt−sds·∫0+∞χijsφ˙ijt−sds−Iijt,Δuijtk=αijkuijtk,i=1,2,…,m,  j=1,2,…,n,  k∈Z.

Obviously, system (15) is equivalent to system (4). So, we investigate the existence, uniqueness, and global exponential stability of almost periodic solution for system (15).

Together with system (15) we consider the linear system

(16) u˙ijt=−dijφtuijt,t≠tk,Δuijtk=αijkuijtk,k∈Z,

where $d_{ij}^{\varphi }(t)≔a_{ij}(\varphi_{ij}(t))b_{ij}(t,\varphi_{ij}(t))/\varphi_{ij}(t)\in [{\underline{\theta }}_{ij},{\bar{\theta }}_{ij}]$, *t* ∈ *ℝ*, where *i* = 1,2,…, *m* and *j* = 1,2,…, *n*.

Now let us consider the equations

(17) $$\begin{array}{c} {\dot{u}}_{ij}\left(\;t\;\right)=-d_{ij}^{\varphi }\left(\;t\;\right)u_{ij}\left(\;t\;\right),\;t_{k-1}<t\leq t_{k}, \end{array}$$

and their solutions

(18) $$\begin{array}{c} u_{ij}\left(\;t\;\right)=u_{ij}\left(\;s\;\right)e^{-\int_{s}^{t}d_{ij}^{\varphi }\left(l\right)dl} \end{array}$$

for *t*_*k*−1_ < *s* < *t* ≤ *t*_*k*_, where *i* = 1,2,…, *m* and *j* = 1,2,…, *n*.

Then [31], the solutions of system (16) are in the form

(19) uijt;t0;uijt0=Wijt,t0uijt0,t0∈R,  i=1,2,…,m,  j=1,2,…,n,

where

(20) Wijφt,s=e−∫stdijφldl,tk−1<s<t<tk;∏l=mk+11+αijle−∫stdijφldl,tm−1<s≤tm<tk<t≤tk+1.

**Lemma 6 (see [6]).**

If the conditions (*H*_3_) and (*H*_6_)-(*H*_7_) and the following condition hold:

($H_{8}$) $\lambda_{ij}≔{\underline{\theta }}_{ij}-N\ln\left(1+\sup_{k\in \mathbb{Z}}\left|\alpha_{ijk}\right|\right)>0$, where constant *N* is determined in Lemma 3, where *i* = 1,2,…, *m* and *j* = 1,2,…, *n*,

then

(21) $$\begin{array}{c} \left|\;W_{ij}^{\varphi }\left(\;t,s\;\right)\;\right|\leq \xi_{ij}e^{-\lambda_{ij}\left(t-s\right)}, \end{array}$$

where *φ* ∈ *𝕏* and *ξ*_*ij*_≔exp⁡{*N*ln⁡(1 + sup_*k*∈*ℤ*_ | *α*_*ijk*_|)}, where *i* = 1,2,…, *m* and *j* = 1,2,…, *n*.

**Lemma 7 .**

If the conditions (*H*_3_) and (*H*_6_)–(*H*_8_) hold, then

(22) $$\begin{array}{c} \left|\;W_{ij}^{\varphi }\left(\;t,s\;\right)-W_{ij}^{\phi }\left(\;t,s\;\right)\;\right|\leq {\bar{\sigma }}_{ij}\xi_{ij}{\left\|\;\varphi -\phi \;\right\|}_{X}e^{-\lambda_{ij}\left(t-s\right)}, \end{array}$$

where *φ*, *ϕ* ∈ *𝕏*, where *i* = 1,2,…, *m* and *j* = 1,2,…, *n*.

**Proof**

By (*H*_3_), we have

(23) e−∫stdijφldl−e−∫stdijϕldl=e−∫stdijζldldijφt−dijϕt=e−∫stdijζldlaijφijtbijt,φijtφijt−aijϕijtbijt,ϕijtϕijt≤e−∫stdijζldlσ¯ijφ−ϕX,

where *d*_*ij*_^*ζ*^ is between *d*_*ij*_^*φ*^ and *d*_*ij*_^*ϕ*^, where *i* = 1,2,…, *m* and *j* = 1,2,…, *n*.

By (20) and (23), we have
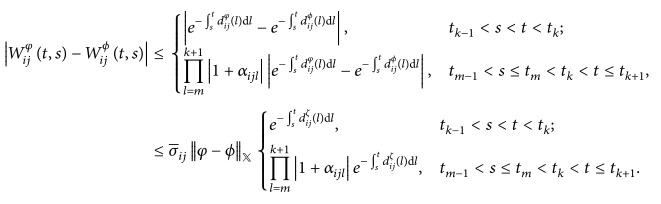
(24)Similar to the proof in Lemma 6, we obtain that

(25) $$\begin{array}{c} \left|\;W_{ij}^{\varphi }\left(\;t,s\;\right)-W_{ij}^{\phi }\left(\;t,s\;\right)\;\right|\leq {\bar{\sigma }}_{ij}\xi_{ij}{\left\|\;\varphi -\phi \;\right\|}_{X}e^{-\lambda_{ij}\left(t-s\right)}, \end{array}$$

where *i* = 1,2,…, *m* and *j* = 1,2,…, *n*. This completes the proof.

## 3. Almost Periodic Solution

In this section, the existence and uniqueness of almost periodic solution of system (4) will be studied.

For any bounded function *f* ∈ *C*(*ℝ*), *f* ^+^ = sup_*s*∈*ℝ*_ | *f*(*s*)|; *f* ^−^ = inf_*s*∈*ℝ*_ | *f*(*s*)|.

Let

(26) K1≔maxi,j⁡ξija¯ijIij+λij−ξijωij,K2≔maxi,j⁡a¯ijIij+λij+ξijθ¯ijλij−λij+ξijθ¯ijωij,ωij≔a¯ij∑Bkl∈Nri,jBijkl+Lijf+a¯ij∑Ckl∈Nri,jCijkl+Mijg,μij≔∑Bkl∈Nri,jBijkl+LijfK+∑Ckl∈Nri,jCijkl+MijgK+Iij+,νij≔∑Bkl∈Nri,jBijkl+Lijf+2∑Ckl∈Nri,jCijkl+LijgK,

where *i* = 1,2,…, *m* and *j* = 1,2,…, *n*.

**Theorem 8 .**

Assume that (*H*_1_)–(*H*_8_) hold; suppose further that

($H_{9}$) $\eta ≔{max}_{\left(i,j\right)}\{\xi_{ij}\omega_{ij}/\lambda_{ij},[1+\xi_{ij}{\bar{\theta }}_{ij}/\lambda_{ij}]\omega_{ij}\}<1$;
($H_{10}$) $\delta ≔{max}_{\left(i,j\right)}\{{\bar{\sigma }}_{ij}K+\left(\xi_{ij}{\bar{\theta }}_{ij}/\lambda_{ij}\right)[(L_{ij}^{a}+{\bar{\sigma }}_{ij}{\bar{a}}_{ij})\mu_{ij}+{\bar{a}}_{ij}\nu_{ij}]+(L_{ij}^{a}\mu_{ij}+{\bar{a}}_{ij}\nu_{ij})\}<1$.

Then system (4) has a unique almost periodic solution.

**Proof**

Define a map Φ on *𝕏* by

(27) Φφt=Φ11φt,…,Φijφt,…,ΦmnφtT,

where

(28) $$\begin{array}{c} \left(\;\Phi_{ij}\varphi \;\right)\left(\;t\;\right)=\overset{t}{\underset{-\infty }{\int }}W_{ij}^{\varphi }\left(\;t,s\;\right)F_{ij}^{\varphi }\left(\;s\;\right)ds, \end{array}$$

in which
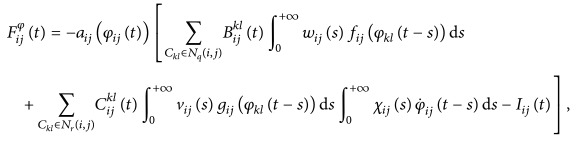
(29)for *i* = 1,2,…, *m*, *j* = 1,2,…, *n*, and *k* ∈ *ℤ*, ∀*t* ∈ *ℝ*.

Let *𝕏* ^*∗*^ be a subset of *𝕏* defined by

(30) X∗=φ∈X:φX≤K,K≔maxK1,K2.

Firstly, we prove that Φ is self-mapping from *𝕏* ^*∗*^ to *𝕏* ^*∗*^.

Similar to the argument as that in Theorem  4.1 of [31], it is easy to prove that Φ*φ* is almost periodic. For *t* ≠ *t*_*k*_, *k* ∈ *ℤ*, we obtain that

(31) Φ˙ijφtddtΦijφt=−dijφtΦijφt+Fijφt,

together with the almost periodicity of Φ_*ij*_ *φ*; ${\dot{\Phi }}_{ij}\varphi$ is also almost periodic, where *i* = 1,2,…, *m* and *j* = 1,2,…, *n*. So Φ*φ* ∈ *𝕏*.

For arbitrary *φ* ∈ *𝕏* ^*∗*^ it follows from (*H*_2_)–(*H*_4_), Lemma 6, and (*H*_9_) that 
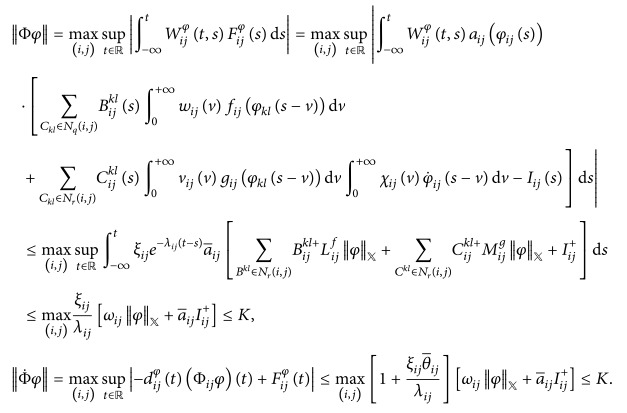
(32)From (32), ‖Φ*φ*‖_*𝕏*_ ≤ *K*, which implies that Φ*φ* ∈ *𝕏* ^*∗*^.

Secondly, we will prove that the mapping Φ is a contraction mapping of *𝕏* ^*∗*^. ∀*φ* = {*φ*_*ij*_}, *ϕ* = {*ϕ*_*ij*_} ∈ *𝕏* ^*∗*^, it follows from (*H*_2_)–(*H*_4_), Lemmas 6 and 7, and (*H*_10_) that

(33) Φφt−Φϕt =maxi,j supt∈R⁡∫−∞tWijφt,sFijφs−Fijϕsds +maxi,j supt∈R⁡∫−∞tWijφt,s−Wijϕt,sFijϕsds ≤maxi,j supt∈R⁡∫−∞tWijφt,s−∑Bkl∈Nri,jaijφijsBijkls ·∫0∞wijιfijφkls−ιdι +∑Bkl∈Nri,jaijϕijsBijkls ·∫0∞wijιfijϕkls−ιdι −∑Ckl∈Nri,jaijφijsCijkls ·∫0∞vijιgijφkls−ιdι ·∫0∞χijιφ˙ijs−ιdι +∑Ckl∈Nri,jaijϕijsCijkls ·∫0∞vijιgijϕkls−ιdι ·∫0∞χijιϕ˙ijs−ιdι +aijφijs−aijϕijsIijs−∑Bkl∈Nri,jaijφijsBijklsds +maxi,j supt∈R⁡∫−∞tσ¯ijξije−λijt−sdsa¯ij ·∑Bkl∈Nri,jBijkl+LijfK+∑Ckl∈Nri,jCijkl+MijgK+Iij+ ·φ−ϕX ≤maxi,j supt∈R⁡∫−∞tξije−λijt−s ·∑Bkl∈Nri,jLijaK+a¯ijBijkl+Lijf +∑Ckl∈Nri,jLijaMijgK+2a¯ijLijgKCijkl++LijaIij+ds ·φ−ϕX +maxi,j⁡σ¯ija¯ijξijλij ·∑Bkl∈Nri,jBijkl+LijfK+∑Ckl∈Nri,jCijkl+MijgK+Iij+ ·φ−ϕX ≤maxi,j⁡ξijλijLijaμij+a¯ijνijφ−ϕX +maxi,j⁡σ¯ija¯ijξijλijμijφ−ϕX ≤maxi,j⁡ξijλijLija+σ¯ija¯ijμij+a¯ijνijφ−ϕX ≤δφ−ϕX,Φ˙φt−Φ˙ϕt =maxi,j supt∈R⁡dijϕtΦijϕt −dijφtΦijφt+Fijφt−Fijϕt ≤maxi,j supt∈R⁡dijφt−dijϕtΦijϕ +dijφtΦijφt−Φijϕt+Fijφt−Fijϕt ≤maxi,j⁡σ¯ijK+ξijθ¯ijλijLija+σ¯ija¯ijμij+a¯ijνij +Lijaμij+a¯ijνijσ¯ijK+ξijθ¯ijλijLija+σ¯ija¯ijμij+a¯ijνijφ−ϕX ≤δφ−ϕX.

By (33), one has

(34) $$\begin{array}{c} {\left\|\;\Phi \left(\;\varphi \;\right)-\Phi \left(\;\phi \;\right)\;\right\|}_{X}\leq \delta {\left\|\;\varphi -\phi \;\right\|}_{X}, \end{array}$$

where *δ* ∈ (0,1). By the contracting mapping principle, there exists a unique fixed point $\varphi^{*}\in \bar{\Omega }$ satisfying Φ(*φ* ^*∗*^) = *φ* ^*∗*^, which implies that system (15) has a unique almost periodic solution *φ* ^*∗*^ with ‖*φ* ^*∗*^‖_*𝕏*_ ≤ *K*. That is, system (4) has a unique almost periodic solution. This completes the proof.

**Remark 9 .**

In recent years, there are many scholars concerned with the almost periodic solution of Cohen-Grossberg neural networks. The main method is the antiderivative method. We consider the following simple Cohen-Grossberg neural networks:

(35) u˙it=−aiuibiui−∑j=1ncijtfjuj−Iit,i=1,2,…,n.

Similar to the arguments as that in [21–25], system (35) can be written as the following system:

(36) x˙it=−dixitxit+∑j=1ncijtfjhi−1xjt+Iit,

where *x*_*i*_ = *h*_*i*_(*u*_*i*_) and *h*_*i*_(*u*_*i*_) is an antiderivative of 1/*a*_*i*_(*u*_*i*_) with *h*_*i*_(0) = 0, where *i* = 1,2,…, *n*. In order to construct a contraction mapping, the authors [21–25] considered the following auxiliary system associated with system (36):

(37) x˙it=−dixitxit+∑j=1ncijtfjhi−1φjt+Iit,i=1,2,…,n,

where *φ* = (*φ*_1_, *φ*_2_,…, *φ*_*n*_)^*T*^ is an arbitrary almost periodic function. Based on system (37) and by using the exponential dichotomy of linear system, the following mapping was established:
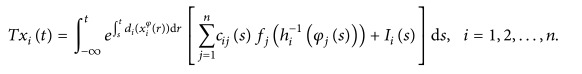
(38)

It is worthwhile to note that system (37) is not a linear auxiliary system. The right side of system (37) is nonlinear about *x*_*i*_ (i.e., *d*_*i*_(*x*_*i*_)*x*_*i*_); then there is nonlinear term about *x*_*i*_ in auxiliary system (37), so the exponential dichotomy of linear system cannot be used. In this paper, we consider system (15) as an equivalent form of system (4). On the right side of system (15), the first term keeps unchangeable; then linear auxiliary system (15) associated with system (4) is obtained. Further, owing to the advent of *d*_*i*_(*x*_*i*_), mapping (38) is not the same as ever. When we verify that *T* is a contraction mapping, the term *e* ^∫_*s*_^*t*^*d*_*i*_(*x*_*i*_^*φ*^(*r*))d*r*^ of (38) must be considered since it depends on *φ* (see (33)). However, the authors in [21–25] ignored this point. Therefore, our work in this paper compensates for the deficiency in papers [21–25]. Clearly, as *a*_*ij*_(*u*_*ij*_) = *a*_*ij*_ and *b*_*ij*_(*t*, *u*_*ij*_) = *u*_*ij*_, where *a*_*ij*_ > 0 is a constant, then the auxiliary system (15) will be changed into the corresponding form in the literature [3–8].

**Remark 10 .**

In view of Theorem 8, we can easily see that the neutral term and impulsive effects bring great difficulty to the proof. And (*H*_8_)–(*H*_10_) in Theorem 8 indicate that the impulsive effect and neutral term have negative effect on the existence and uniqueness of almost periodic solution of system (4). The work of this paper extends the main results in [3–8].

## 4. Global Exponential Stability of Almost Periodic Solution

In this section, we study global exponential stability of almost periodic solution of system (4) by constructing a suitable Lyapunov functional.

For convenience, let

(39) τij≔∑Bkl∈Nri,jBijkl+MijfLija+∑Ckl∈Nri,j2KCijkl+MijgLija+LijaIij+,ρij≔∑Bkl∈Nri,ja¯ijBijkl+Lijf+∑Ckl∈Nri,j2Ka¯ijCijkl+Lijg,σij≔∑Ckl∈Nri,ja¯ijCijkl+Mijg,

where *K* is defined as that in Theorem 8, where *i* = 1,2,…, *m* and *j* = 1,2,…, *n*.

**Theorem 11 .**

Assume that (*H*_1_)–(*H*_10_) hold; suppose further that

($H_{11}$) sup_(*i*,*j*,*k*)_ | 1 + *α*_*ijk*_ | ≤1;
($H_{12}$) $(1-\sigma_{ij}){\underline{\theta }}_{ij}>\sigma_{ij}{\underline{\theta }}_{ij}+\tau_{ij}+\rho_{ij}$, where *i* = 1,2,…, *m* and *j* = 1,2,…, *n*.

Then system (4) has a unique almost periodic solution, which is globally exponentially stable.

**Proof**

It follows from Theorem 8 that system (4) has a unique almost periodic solution *y* = {*y*_*ij*_} with initial value *ϕ* = {*ϕ*_*ij*_}. We next show that the almost periodic solution *y* is globally exponentially stable.

Make a transformation for system (4): *x*_*ij*_ = *z*_*ij*_ − *y*_*ij*_, where *i* = 1,2,…, *m* and *j* = 1,2,…, *n*, where *z* = {*z*_*ij*_} is arbitrary solution of system (4) with initial value *ψ* = {*ψ*_*ij*_}.

By (5) and (*H*_11_), there exist small enough positive constants *ω* and *ϵ* such that

(40) ∫0∞wijseωsds≤1+ϵ,∫0∞vijseωsds≤1+ϵ,∫0∞χijseωsds≤1+ϵ,ω−θ_ij−1−ϵθ¯ij+θ_ijσij−τij−1−ϵρij1−1−ϵσij<0,

where *i* = 1,2,…, *m* and *j* = 1,2,…, *n*.

Define

(41) $$\begin{array}{c} V_{ij}\left(\;t\;\right)=e^{\omega t}\left|\;x_{ij}\left(\;t\;\right)\;\right|,\;i=1,2,\ldots ,m,\;\;j=1,2,\ldots ,n. \end{array}$$

In view of system (4), for *t* ≠ *t*_*k*_, *k* ∈ *ℤ*, we have

(42) D+Vijt≤ωeωtxijt+eωtsgn⁡xijtx˙ijt≤ωeωtxijt+eωt−aijzijtbijt,zijt−aijyijtbijt,yijtsgn⁡zijt−yijt+aijzijt∑Bkl∈Nri,jBijklt·∫0∞wijsfijzklt−sds−aijyijt·∑Bkl∈Nri,jBijklt∫0∞wijsfijyklt−sds+aijzijt∑Ckl∈Nri,jCijklt·∫0∞vijsgijzklt−sds·∫0∞χijsz˙ijt−sds−aijyijt·∑Ckl∈Nri,jCijklt∫0∞vijsgijyklt−sds·∫0∞χijsy˙ijt−sds+aijzijt−aijyijtIijt≤ωeωtxijt−θ_ij−∑Bkl∈Nri,jBijkl+MijfLija−∑Ckl∈Nri,j2KCijkl+MijgLija−LijaIij+eωtxijt+∑Bkl∈Nri,ja¯ijBijkl+Lijf∫0∞eωswijs·eωt−sxklt−sds+∑Ckl∈Nri,ja¯ijCijkl+Mijg·∫0∞eωsχijseωt−sx˙ijt−sds+∑Ckl∈Nri,j2Ka¯ijCijkl+Lijg∫0∞eωsvijs·eωt−sxklt−sds≤ω−θ_ij+∑Bkl∈Nri,jBijkl+MijfLija+∑Ckl∈Nri,j2KCijkl+MijgLija+LijaIij+Vijt+1−ϵ∑Bkl∈Nri,ja¯ijBijkl+Lijf+∑Ckl∈Nri,j2Ka¯ijCijkl+Lijgsups∈−∞,t⁡Vkls+1−ϵ·∑Ckl∈Nri,ja¯ijCijkl+Mijgsups∈−∞,t⁡eωsx˙ijs≤ω−θ_ij+τijVijt+1−ϵρijsups∈−∞,t⁡Vkls+1−ϵσij·sups∈−∞,t⁡eωsx˙ijs,i=1,2,…,m,  j=1,2,…,n.

Further, for *t* ≠ *t*_*k*_, *k* ∈ *ℤ*, we obtain from system (4) that

(43) eωtx˙ijt=eωtz˙ijt−y˙ijt≤eωtaijzijtbijt,zijt−aijyijtbijt,yijtzijt−yijt·xijt+eωtaijzijt∑Bkl∈Nri,jBijklt·∫0∞wijsfijzklt−sds−aijyijt∑Bkl∈Nri,jBijklt·∫0∞wijsfijyklt−sds+eωtaijzijt·∑Ckl∈Nri,jCijklt∫0∞vijsgijzklt−sds·∫0∞χijsz˙ijt−sds−aijyijt∑Ckl∈Nri,jCijklt·∫0∞vijsgijyklt−sds∫0∞χijsy˙ijt−sds+eωtaijzijt−aijyijtIijt≤θ¯ij+∑Bkl∈Nri,jBijkl+MijfLija+∑Ckl∈Nri,j2KCijkl+MijgLija+LijaIij+·eωtxijt+∑Bkl∈Nri,ja¯ijBijkl+Lijf∫0∞eωswijs·eωt−sxklt−sds+∑Ckl∈Nri,ja¯ijCijkl+Mijg·∫0∞eωtχijsx˙ijt−sds+∑Ckl∈Nri,j2Ka¯ijCijkl+Lijg·∫0∞eωsvijseωt−sxklt−sds≤θ¯ij+∑Bkl∈Nri,jBijkl+MijfLija+∑Ckl∈Nri,j2KCijkl+MijgLija+LijaIij+·Vijt+∑Bkl∈Nri,ja¯ijBijkl+Lijf∫0∞eωswijsVklt−sds+∑Ckl∈Nri,ja¯ijCijkl+Mijg∫0∞eωsχijseωt−sx˙ijt−sds+∑Ckl∈Nri,j2Ka¯ijCijkl+Lijg∫0∞eωsvijsVklt−sds≤θ¯ij+τijsups∈−∞,t⁡Vijs+1−ϵρijsups∈−∞,t⁡Vkls+1−ϵσij·sups∈−∞,t⁡eωsx˙ijs,

which implies that

(44) sups∈−∞,t⁡eωsx˙ijs≤θ¯ij+τij1−1−ϵσijsups∈−∞,t⁡Vijs+1−ϵρij1−1−ϵσijsups∈−∞,t⁡Vkls,

where *i* = 1,2,…, *m* and *j* = 1,2,…, *n*. Substituting (44) into (42) leads to

(45) D+Vijt≤ω−θ_ij+τijVijt+1−ϵρijsups∈−∞,t⁡Vkls+1−ϵ2ρijσij1−1−ϵσijsups∈−∞,t⁡Vkls+1−ϵθ¯ij+τijσij1−1−ϵσijsups∈−∞,t⁡Vijs,t≠tk,  k∈Z,

where *i* = 1,2,…, *m* and *j* = 1,2,…, *n*.

For *t* ≤ 0, note that

(46) Vijt=eωtxijt≤Pψ−ϕ∞,i=1,2,…,m,  j=1,2,…,n,

where *P* > 1. Next, we claim that

(47) Vijt≤Pψ−ϕ∞,∀t∈0,+∞,  i=1,2,…,m,  j=1,2,…,n.

From (*H*_7_), we observe that

(48) xijtk+1+αijkzijtk−yijtk≤xijtktk,k∈Z,

where *i* = 1,2,…, *m* and *j* = 1,2,…, *n*. By way of contradiction, assume that (47) does not hold. Then, there must exist *i*_0_ ∈ {1,2,…, *m*}, *j*_0_ ∈ {1,2,…, *n*}, and *t*_0_ ∈ [0, +*∞*∖{*t*_*k*_} such that

(49) Vi0j0t0=Pψ−ϕ∞,D+Vi0j0t0>0,Vijt≤Pψ−ϕ∞,∀t∈−∞,t0,  i=1,2,…,m,  j=1,2,…,n.

By (45), we have from (40) that

(50) 0<D+Vi0j0t0≤ω−θ_i0j0+τi0j0Vi0j0t0+1−ϵ·ρi0j0sups∈−∞,t0Vk′l′s+1−ϵ2ρi0j0σi0j01−1−ϵσi0j0sups∈−∞,t0⁡Vk′l′s+1−ϵθ¯i0j0+τi0j0σi0j01−1−ϵσi0j0sups∈−∞,t0⁡Vi0j0s≤ω−θ_i0j0+τi0j0+1−ϵρi0j0+1−ϵ2ρi0j0σi0j01−1−ϵσi0j0+1−ϵθ¯i0j0+τi0j0σi0j01−1−ϵσi0j0Pψ−ϕ∞=ω−θ_i0j0−1−ϵθ¯i0j0+θ_i0j0σi0j0−τi0j0−1−ϵρi0j01−1−ϵσi0j0·Pψ−ϕ∞<0.

This is a contradiction. So our claim is valid. Therefore,

(51) $$\begin{array}{c} \overset{m}{\underset{i=1}{\sum }}\;\overset{n}{\underset{j=1}{\sum }}\left|\;x_{ij}\left(\;t\;\right)\;\right|\leq mnP{\left\|\;\psi -\phi \;\right\|}_{\infty }e^{-\omega t},\;\forall t>0. \end{array}$$

Thus, the almost periodic solution of system (4) is globally exponentially stable. This completes the proof.

**Remark 12 .**

If system (4) satisfies (*H*_7_), the proof of Theorem 11 indicates that the impulses have no effect on the global exponential stability of the system. From condition (*H*_11_) in Theorem 11, we can easily see that the neutral terms have negative effect on the global exponential stability of almost periodic solution of system (4). In Theorem 11, (44) is crucial. If there is no neutral term ${\dot{x}}_{ij}$ in the right side of system (4), (44) is not needed (see papers [3–8, 21–24]). Because of the presence of the neutral term ${\dot{x}}_{ij}$, we have to get (44). The effect of (44) is to eliminate ${\dot{x}}_{ij}$ in *D* ^+^ *V*_*ij*_ (see (45)). In [3–8, 26], the (almost) periodic dynamics of many special cases of system (4) has been considered. We also generalize the main results of [3–8, 26].

**Remark 13 .**

In [47, 48], the authors studied the global exponential stability of (pseudo) almost periodic solutions for CNNs with leakage delays, which can be transformed into the neutral systems. Theorem 11 gives a possible method to study the global exponential stability of almost periodic solutions of the neutral systems and the method differs from that in [47, 48]. By using the method in this paper, we could obtain new criteria for the global exponential stability of almost periodic solutions of CNNs with leakage delays, which supplements the corresponding result in [47, 48].

## 5. An Example and Numerical Simulations

**Example 1 .**

Consider the following neutral-type Cohen-Grossberg SICNNs with distributed delays:
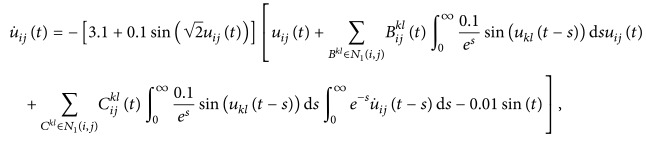
(52)where *i*, *j* = 1,2,

(53) B11sB12sB21sB22s=C11sC12sC21sC22s=0.1sin⁡3s0.3cos⁡3s0.2sin⁡3s0.1cos⁡3s,∀s∈R.

**Figure 1:**
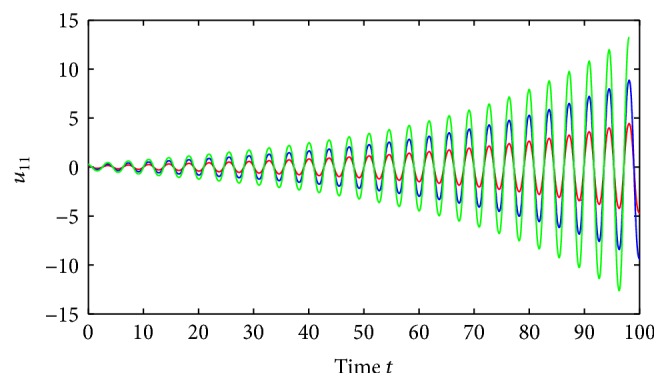
Unstability of state variables *u*_11_ of system (52).

**Figure 2:**
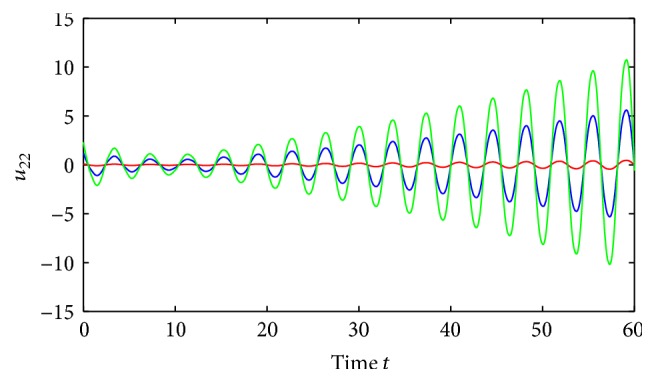
Unstability of state variables *u*_22_ of system (52).

By using the MATLAB dde23, Figures 1 and 2 depict the time responses of state variables (*u*_11_, *u*_22_)^*T*^ in system (52) with step 0.01, respectively. It is easy to see that system (52) is not stable.

Consider system (52) with impulses:
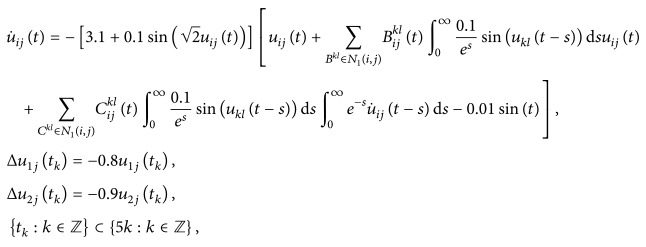
(54)where *i*, *j* = 1,2.

Corresponding to system (4), ${\underline{a}}_{ij}=3$, ${\bar{a}}_{ij}=3.2$, ${\underline{b}}_{ij}={\bar{b}}_{ij}=1$, *w*_*ij*_(*s*) = *v*_*ij*_(*s*) = *χ*_*ij*_(*s*) = *e* ^−*s*^, *f*_*ij*_(*s*) = *g*_*ij*_(*s*) = 0.1sin⁡(*s*), and *I*_*ij*_(*s*) = 0.01sin⁡(*s*), where *i*, *j* = 1,2 and *s* ∈ *ℝ*. Clearly, we have *N* = 1,

(55) ∑Bkl∈N11,1B11kl+=∑Ckl∈N11,1C11kl+=0.7,∑Bkl∈N11,2B12kl+=∑Ckl∈N11,2C12kl+=1.4,∑Bkl∈N12,1B21kl+=∑Ckl∈N12,1C21kl+=1,∑Bkl∈N12,2B22kl+=∑Ckl∈N12,2C22kl+=1.8.

By an easy calculation, we obtain ${\bar{\theta }}_{ij}=3.2$, ${\bar{\sigma }}_{ij}=0.1$, ${\underline{\theta }}_{ij}=3$, *λ*_1*j*_ = 2.4, *λ*_2*j*_ = 2.32, *ξ*_1*j*_ = 1.66, *ξ*_2*j*_ = 1.87, *i*, *j* = 1,2, and

(56) η≈0.22,δ≈0.35.

Further, we also have that

(57) $$\begin{array}{c} \left(\;1-\sigma_{ij}\;\right){\underline{\theta }}_{ij}-\sigma_{ij}{\underline{\theta }}_{ij}+\tau_{ij}+\rho_{ij}>0.02,\;i,j=1,2. \end{array}$$

It is easy to verify that all the conditions of Theorem 11 are satisfied and system (54) has a unique almost periodic solution, which is globally exponentially stable.

**Figure 3:**
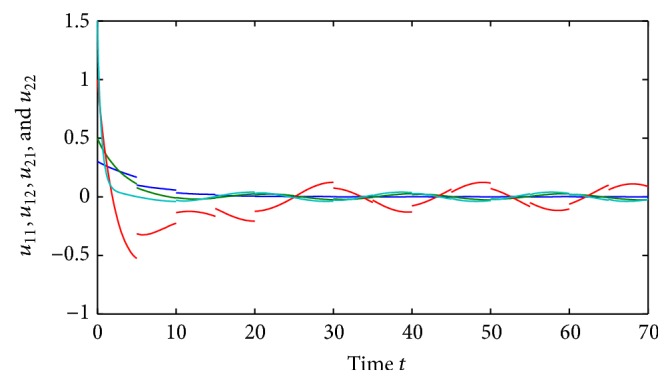
Almost periodicity of state variables of system (52).

**Figure 4 fig4:**
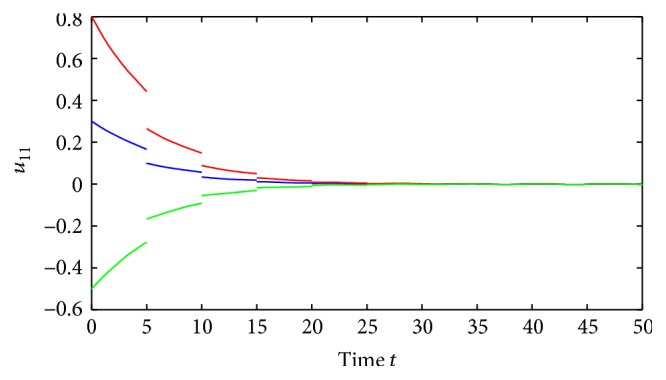
Exponential stability of state variables *u*_11_ of system (52).

**Figure 5 fig5:**
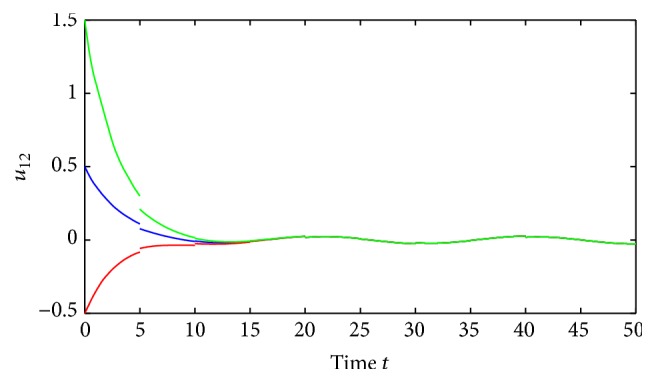
Exponential stability of state variables *u*_12_ of system (52).

**Figure 6 fig6:**
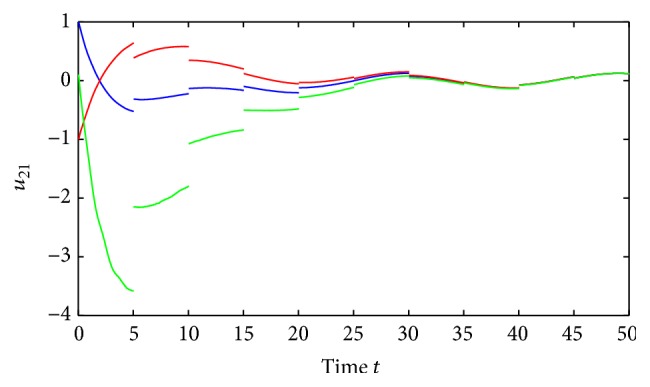
Exponential stability of state variables *u*_21_ of system (52).

**Figure 7:**
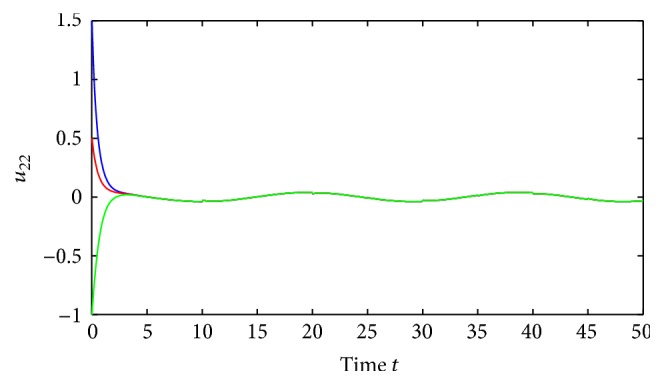
Exponential stability of state variables *u*_22_ of system (52).

Also, by utilizing the MATLAB dde23, Figures 3 [Fig fig4] [Fig fig5] [Fig fig6]–7 depict the time responses of state variables (*u*_11_, *u*_12_, *u*_21_, *u*_22_)^*T*^ in system (54) with step 0.01, respectively. It confirms that the proposed condition in Theorem 11 leads to globally exponentially stable almost periodic solution for system (54).

**Remark 14 .**

Example 1 shows that the model without impulses is not stable, but it will be stable in the case with impulses.

## 6. Discussion

In this paper, the neutral Cohen-Grossberg shunting inhibitory cellular neural networks with distributed delays and impulses are considered. By employing fixed point theory and constructing suitable Lyapunov functional, some new sufficient conditions are obtained for the existence and global exponential stability of almost periodic solution of the system. Conditions (*H*_8_)–(*H*_11_) in Theorems 8 and 11 indicate that the neutral terms and impulsive effects have negative effect on the existence, uniqueness, and global exponential stability of almost periodic solution of the neutral-type system. However, if system (4) satisfies (*H*_7_), Theorem 11 indicates that the impulses have no effect on the global exponential stability of the system. The method used in this paper provides a possible method to study the existence, uniqueness, and global exponential stability of almost periodic solution of other neutral neural networks with impulsive effects.
